# Lipoprotein(a) and inflammation in patients with atrial fibrillation after electrical cardioversion

**DOI:** 10.1186/1477-5751-10-15

**Published:** 2011-11-12

**Authors:** Franjo Naji, Miso Sabovic

**Affiliations:** 1 Department of Cardiology and Angiology, University Clinical Centre, Maribor, Slovenia; 2 Department of Vascular Diseases, University Clinical Centre, Ljubljana, Slovenia

**Keywords:** atrial fibrillation, lipoprotein(a), cardioversion, inflammation

## Abstract

**Background:**

Recently few studies tried to confirm the association between AF and lipoprotein(a) (Lp(a)), however the results remained conflicted. In present study we evaluated the possible interaction between Lp(a), inflammatory state and echocardiographic characteristics in patients after successful electrical cardioversion (EC) of persistent AF. We also tried to investigate the role of Lp(a) as a possible prognostic factor for AF recurrence after successful EC.

**Results:**

Data of 79 patients admitted due to planned EC was analyzed. After successful procedure patients were monitored for 2 years. For analytical purposes patients were divided in two groups according to AF recurrence. There was no significant difference between Lp(a) levels in both groups. We also didn't find any positive correlation between Lp(a) and CRP levels, as well as between Lp(a) levels and left atrium diameter. For logistic and survival analysis optimal cut-off value of Lp(a) ≥ 0.32 (upper quartile) was used. In logistic regression model with AF recurrence as dependent variable Lp(a) didn't show any statistically significant association with AF recurrence. Survival analysis showed slightly higher AF recurrence rate in group with higher Lp(a) levels but not to the level of statistical significance (log rank test, *p *= 0.62).

**Conclusions:**

We weren't able to confirm the association between Lp(a) levels and AF recurrence, inflammation and left atrium diameter in patients after successful EC of persistent AF. Further studies are needed to elucidate the role of Lp(a) in this clinical setting.

## Introduction

Atrial fibrillation (AF) is one of major causes for morbidity and hospitalization [1]. It is also important risk factor for thrombembolic complications and cerebrovascular disease[2]. Existing data strongly support its connection with ishemic heart disease, arterial hypertension, heart failure, obesity and metabolic syndrome [3-7]. In past years few studies tried to elucidate the association between AF and lipoprotein(a), particularly in connection with thrombembolic complications [8,9]. Major component of lipoprotein(a), or Lp(a) is large glycoprotein molecule named apoprotein(a), which is produced in hepatocytes. It binds with apo B100 component of LDL to form Lp(a). It was suggested that apoprotein(a) because of its homology to plasminogen interferes with fibrinolytic system and hence modulates thrombogenic activity in plasma [10]. Concomitantly in AF patients extensive inflammation takes place, which interferes with coagulation as well as oxidation [11]. It was also shown that levels of Lp(a) correlate with inflammatory state and coagulation disorders [12]. However a connection between Lp(a) and AF could not be established [13]. In this study we evaluated the possible interaction between Lp(a), cholesterol levels and inflammatory state in patients after successful electrical cardioversion (EC) of persistent AF. We also tried to investigate the role of Lp(a) as a possible prognostic factor for AF recurrence after successful EC.

## Results

85 consecutive patients were included in analysis. We lost 6 patients in follow-up period, so finally data of 79 patients was analyzed. Patients were followed for 2 years. Baseline characteristics are displayed in Table 1. In group of patients with recurrent AF we observed a significantly prolonged AF duration period prior to EC. In group with sinus rhythm we observed a greater proportion of patients treated with amiodarone, but not to the level of statistical significance. There were no other major differences between both groups, including Lp(a) levels. We also didn't find any positive correlation between Lp(a) and CRP levels, as well as between Lp(a) levels and left atrium diameter. For purposes of univariate, multivariate and survival analysis optimal cut-off value of Lp(a) ≥ 0.32 (upper quartile) was used. In logistic regression model with AF recurrence as dependent variable and Lp(a), CRP, age, sex, duration of AF, arterial hypertension, diabetes, ischemic heart disease, echocardiographic measurements and concomitant medications as independent covariates, Lp(a) didn't show any statistically significant association with AF recurrence.

**Table 1:** Patients characteristics

	All patients (n = 79)	AF recurrence (n = 47)	Sinus rhythm (n = 32)	*P *value
Duration of AF	6.3 ± 9.3	8.0 ± 11.5	3.8 ± 3.0	<0.05

Age	62 ± 12	61 ± 14	64 ± 10	0.38

Sex	53 (67.1%)	35 (74.5%)	18 (56.3%)	0.14

Ishemic heart disease	9 (11.4%)	4 (8.5%)	5 (15.6%)	0.27

Arterial hypertension	58 (73.4%)	36 (76.6%)	22 (68.8%)	0.45

Diabetes	3 (3.8%)	0	3 (9.4%)	0.06

Treatment				

- Amiodarone	60 (75.9%)	32 (68.1%)	28 (87.5%)	0.06

- Propafenone	19 (24.1%)	13 (27.7%)	6 (18.8%)	0.43

- Beta blocker	23 (29.1%)	17 (36.2%)	6 (18.8%)	0.13

-Angiotensin receptor blocker	5 (6.3%)	3 (6.4%)	2 (6.3%)	1.0

- ACE inhibitor	51 (64.4%)	32 (68.1%)	19 (59.4%)	0.48

- Statin	20 (25.3%)	9 (19.1%)	11 (34.4%)	0.19

Cholesterol levels (mmol/L)	5.1 ± 0.9	5.3 ± 1.1	4.9 ± 0.6	0.22

CRP (mg/L)	5.6 ± 7.4	5.1 ± 6.6	6.2 ± 8.6	0.61

Lp(a) (g/L)	0.21 ± 0.24	0.22 ± 0.23	0.19 ± 0.25	0.61

Ejection fraction (%)	51 ± 8	52 ± 8	50 ± 10	0.47

Mitral regurgitation (stage 2 or higher)	14 (17.7%)	11 (23.4%)	3 (9.4%)	0.14

Left atrium dimension	5.2 ± 0.8	5.4 ± 0.7	5.1 ± 1.0	0.20

**Figure 1:**
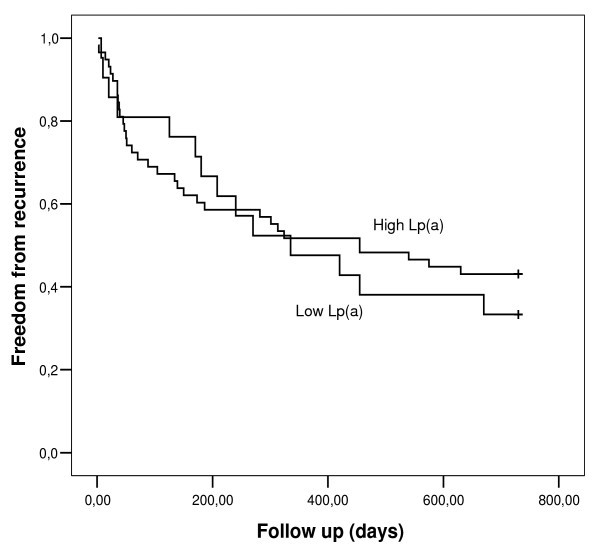
**Kaplan-Meier survival curves according to Lp(a) levels (*p *= 0.62; log rank test)**.

Survival analysis showed slightly higher AF recurrence rate in group with higher Lp(a) levels but not to the level of statistical significance (log rank test, *p *= 0.62; Figure 1).

## Discussion

In present study we tried to evaluate the possible connection between plasma Lp(a) levels and AF. Despite relatively large study population and long observation period we didn't find any positive correlation that would confirm the role of Lp(a) in connection to AF. We also didn't find any correlation between Lp(a) levels and inflammatory status in our patients. After performing survival analysis we found only slightly higher AF recurrence rate in group of patients with higher Lp(a) levels, but not to the level of statistical significance.

Our results are similar to conclusions of previous study conducted by Diaz-Peromingo et al., who also didn't find any relationship between Lp(a) levels and AF [13]. They compared 101 patients with AF and 101 patients with sinus rhythm in control group. They found a higher total cholesterol/HDL ratio in group of patients with AF, however there were no differences between both groups when comparing levels of Lp(a). They concluded that at least in part their results were influenced by ethnic characteristics, since data exists that L(p)a levels are higher in Afro-American and Hispanic population when compared to Caucasians.

Many studies have tried to evaluate the role of Lp(a) in different clinical settings. It was proven that higher levels of Lp(a) are connected with cerebral ischemia and cerebrovascular disease [14]. Igarashi et al. concluded in their study that patients with AF and left atrial thrombus had higher levels of Lp(a) [9]. This could partly be explained by hypothesis that apoprotein(a) due to its similarity with plasminogen interferes with fybrinolitic system [10]. Unfortunately in our study we weren't able to follow coagulation parameters and clinical events, what could give further useful information about the role of Lp(a) in patients with AF. We also weren't able to prove the association between Lp(a) and inflammation, even though there is data that this connection exists [12,15]. On the other hand this could to some extent explain the lack of any significant effect of Lp(a) in our patients. It is possible that in patients with higher level of inflammation there would be stronger association between AF and levels of Lp(a). We also found significantly prolonged duration time of AF prior to EC in our group with recurrent AF. It is possible that this could have a certain impact on our results. Since it was already stated that time to cardioversion predicts the recurrence rate after successful EC, perhaps in different clinical settings the role of Lp(a) could be clarified to greater extent.

## Conclusions

In present study we weren't able to confirm any association between Lp(a) levels and AF recurrence, inflammation and left atrium diameter in patients after successful EC of persistent AF. Further studies are needed to elucidate the role of Lp(a) in this clinical setting, preferably in patients with higher level of inflammation or developed atherosclerosis.

## Methods

This is a retrospective study of patients admitted for planned EC between January 2003 and December 2003. Data collection from hospital's documentation database was made accordingly to hospital ethics policy. Inclusion criteria were AF lasting more than 7 days and successful EC. Patients were excluded, if they had thyroid dysfunction, chronic inflammatory condition (like rheumatoid arthritis), implanted heart valve, pacemaker or cardioverter-defibrillator. They were also excluded if they had previous radiofrequency ablation of atrial arrhythmia.

Anticoagulant treatment was prescribed according to current guidelines and antiarrhythmic therapy was given according to clinician's decision. After admittance patients were fasted overnight. Blood sample was then drawn from antecubital vein. Electrical cardioversion was performed under sedation with propofol. After successful procedure patients were monitored for another 24 hours. Patients where AF recurrence was noticed within 24 hours were excluded from further analysis. Follow-up consisted from regular visits in our outpatient clinic every 1 to 6 months. 12-lead ECG was then recorded, history was taken and clinical examination was performed. In case of symptoms suggestive of AF recurrence 24 hour Holter was recorded. Patients were also advised to visit emergency department in case of palpitations. Documented AF was considered as a study end point.

Patients were divided in two groups according to AF recurrence. Continuous variables between both groups were compared using *t *test and categorical variables with chi square test. For correlation analysis Spearman's rank correlation coefficient was used. We divided Lp(a) levels in quartiles in order to assess the discrimination ability of Lp(a) for AF recurrence. Logistic regression model was used to assess predictors of AF recurrence. For survival analysis we used the Kaplan-Meier method with log rank test. Continuous variables are expressed as mean ± SD and categorical as percentages. A p value of < 0.05 was considered for statistically significant. Statistical analysis was performed using the SPSS 13.0 statistical package (SPSS; Chicago, IL).

## Competing interests

The authors declare that they have no competing interests.

## Authors' contributions

FN participated in design of the study, carried out data collection and statistical analysis and participated in manuscript preparation. MS participated in the design of the study and in the manuscript preparation and revision. All authors read and approved the final manuscript.
